# EUPopLink Country report - Czechia

**DOI:** 10.12688/openreseurope.23458.1

**Published:** 2026-07-16

**Authors:** Vladimír Naxera, Alexandra Halounová, Petia Gueorguieva

**Affiliations:** 1 University of West Bohemia in Pilsen, Pilsen, Plzeň Region, Czech Republic; 2 New Bulgarian University, Sofia, Bulgaria

**Keywords:** populism; euroscepticism; Czechia

## Abstract

This country report examines the relationship between populism and Euroscepticism in the Czech Republic, a country characterised by strong populist parties and a long-standing critical attitude towards the European Union. Using the EUPopLink framework, the report demonstrates that Euroscepticism is prevalent across the political spectrum, not just among explicitly populist parties. Although hard Eurosceptic positions exist, including calls for ‘Czexit’, they remain politically marginal. The findings reveal that the relationship between populism and Euroscepticism is not straightforward: non-populist actors may also adopt Eurosceptic stances, and populist parties exhibit varying degrees and forms of EU criticism. Nevertheless, recent developments suggest that right-wing and Eurosceptic forms of populism are strengthening.

## Introduction

Just as Italy is often referred to as the ‘promised land of populism’ ( Tarchi, 2015), the same can be said for the Czech Republic, particularly due to the variability and strength of populist actors in contemporary Czech politics. For example, in the 2025 elections, populist parties won roughly 55% of the vote. However, unlike Italy, where the Eurosceptic turn is a recent phenomenon, a critical attitude towards the EU has been ever-present in Czech politics and society ( Sychra & Kratochvíl, 2021). Since the Czech Republic joined the EU in 2004, there has always been a certain backlash against the EU. The number of citizens critical of membership remains relatively high (according to some opinion polls, dissatisfaction with membership reached more than 30% in some years and even more than 40% in 2012; cf. CVVM, 2023). According to the Eurobarometer 2025 survey, Czechs currently have the least favorable perception of the European Union among all member states, with only 35% having a positive view of the EU ( EB, 2025). Eurosceptic voices among the parties and the public were strengthened, particularly by the European refugee crisis, despite the Czech Republic’s relative avoidance of it. However, the debate about the EU migration policy has portrayed the EU negatively as an interfering superpower, an image that persists in public discourse.

At the party level, Eurosceptic rhetoric at the time of accession was associated primarily with the far-right Republicans (SPR-RSČ), the Communists of the KSČM, and the conservative Civic Democratic Party (ODS), although from ideologically distinct positions ( Kopecký, 2004). The ODS, although not populist, has been the most stable Eurosceptic voice since the early 1990s (cf. Hanley, 2004). However, it is worth noting that the entire Czech party spectrum has shifted to a more Eurosceptic stance over the years ( Sychra & Kratochvíl, 2021). While several parties take a generally Eurosceptic position, including the politically dominant ANO movement, others support complete withdrawal or a referendum on the issue, such as the far-right party Freedom and Direct Democracy (SPD) and the Communist Party of Bohemia and Moravia (KSČM).

The present country report was prepared as part of the EUPopLink COST Action ( Andreadis & Vasilopoulou, 2026) following the EUPopLink Theoretical Framework and guidelines ( Lorimer et al., 2026).

## Populist political actors

Leaving aside political parties that were only electorally successful in the past (SPR-RSČ from 1992 to 1998 and Public Affairs from 2010 to 2013), three populist parties are currently present in the Chamber of Deputies – Action of Dissatisfied Citizens (ANO), Freedom and Direct Democracy (SPD), and Motorists for Themselves (AUTO), which entered the lower house of the Parliament after this year’s elections. On the other hand, Enough! (Stačilo!) narrowly missed the 5% threshold and have not received any seats.

**Action of Dissatisfied Citizens** (Akce nespokojených občanů; ANO): ANO was founded in 2011 by Andrej Babiš, then one of the richest Czech entrepreneurs, who styled himself as an ordinary citizen and businessman who resented corrupt and incompetent politicians. Babiš proposed apolitical solutions, combining expertise with a populist appeal to ordinary people (cf. Buštíková & Guasti, 2019). From 2013 to 2017, ANO was a junior coalition partner, and from 2017 to 2021, Andrej Babiš served as the PM of a minority government supported by the communist vote. In terms of classification, Andrej Babiš (or the whole party, which is strongly personalized) tends to be described as a valence populist. Over the years, ANO has gradually focused on attracting voters from various other parties. To do so, it changed its rhetoric and shifted from its original valence populism to far-right positions (even though the core of its electorate is made up of former voters of the left-wing Social Democracy). It has strengthened its anti-immigration, nativist, and conservative-nationalist rhetoric (cf. Naxera, 2025a). This shift was accompanied by a change in its faction in the European Parliament, where it now collaborates with several far-right parties within the Patriots for Europe. Currently, ANO is the strongest entity with a stable support of over 30% of the vote, as confirmed by the 2025 parliamentary elections, where ANO convincingly won with just under 35% of the vote.

**Freedom and Direct Democracy** (Svoboda a přímá demokracie; SPD): Led by Tomio Okamura, the SPD is a typical example of the populist radical right. In 2013, independent senator Tomio Okamura founded a movement called Dawn of Direct Democracy (Úsvit přímé demokracie; Úsvit), which successfully entered the lower house of the Parliament that same year. During this term, Okamura was expelled from the party and founded the SPD, which has been part of the Czech party system since the 2017 elections and has its main support base in structurally disadvantaged regions ( Maškarinec & Novotný, 2024). The party is strongly anti-immigrant, and in August 2025, charges were brought against the party and Okamura for inciting hatred in connection with the party’s campaign for the 2024 European Parliament elections. In the European Parliament, SPD is a member of the newly established Europe of Sovereign Nations faction. As for the 2025 elections, although support for the SPD and its coalition partners (a few smaller radical-right parties) remained stable at over 10% in pre-election polls, the party ultimately settled for 7.8%.

**The Motorists for Themselves** (Motoristé sobě; AUTO): was originally a local Prague party, subsequently changing its leadership and name several times. After Petr Macinka, who is associated with the circles around the former president, PM, and chairman of the Civic Democratic Party, Václav Klaus, took over the party, the Motorists became a typical representative of right-wing populism. Motorists for Themselves gained political traction through a tactical alliance with the Oath, a populist party established before the 2021 parliamentary elections by former elite detective Róbert Šlachta. The Oath narrowly missed the 5% threshold in 2021. Despite their different histories and ideologies, the parties joined forces for the 2024 EP elections. The coalition exceeded expectations in the EP elections, finishing third nationally and securing two MEP seats, which joined the Patriots for Europe faction. ^1^ However, before the 2025 parliamentary elections, this coalition collapsed at the instigation of Motorists for Themselves, who voted overwhelmingly against it at the party congress. Both parties thus entered the race for parliamentary seats independently, and in the end, the Motorists came out on top, gaining 6.8% of the votes; meanwhile, Oath received just 1% of the vote, falling once again below the 5% threshold.

**Enough!** (Stačilo!): The Enough! movement was founded in 2025, with members of the KSČM, the Czech Social Democrats (SOCDEM), and a few smaller far-right parties and conspiracy circles running on its candidate lists. Formally, Enough! is not a coalition, which allowed them to avoid the higher threshold for coalitions in the 2025 parliamentary elections. Previously, in the 2024 EP elections, the KSČM and a few smaller parties ran under a coalition with the same name. They won two seats and remained non-attached to any factions in the EP. Overall, Enough! is difficult to classify because it combines the national-conservative and radical-left (KSČM) with several far-right and populist entities. Pro-Russian and anti-European positions are typical (cf. Wondreys et al., 2025), making the grouping very different from many populist radical left parties in Europe.

## Positions on ‘Europe’ and the populism-Euroscepticism nexus

Euroscepticism has been a prominent feature of the Czech political mainstream for a considerable time ( Macek, 2025). We encounter representatives of both soft and hard Euroscepticism, as defined by Taggart & Szczerbiak (2013). Although the latter are not very common in EU member states in the post-Brexit era, there are Czech populist parties, including the far-right SPD and Enough! movement that supports leaving the EU. Several other populist parties oppose a complete withdrawal from the EU but remain critical of its functioning, making them cases of soft Euroscepticism (e.g., ANO and Motorists).

After its founding in 2011, ANO was a rather pro-European party. However, the party’s stance on the EU has evolved over time. For the domestic audience, Babiš began criticizing the EU and its “detached elites” while gradually stressing the importance of sovereignty in handling the migration crisis and, lately, environmental policy. Despite the rhetoric used, ANO remained a constructive player in the EU (cf. Sychra & Kratochvíl, 2021). In conclusion, ANO represents a typical case of soft Euroscepticism. Despite its criticism of the EU’s functioning and its emphasis on necessary changes (e.g., the Green Deal, migration policy), ANO clearly states that the EU, as a union of sovereign states, is crucial for the Czech Republic. Even before the 2025 elections, Babiš repeatedly stated that a referendum on leaving the EU was not on the table. Ultimately, EU membership remains pragmatically advantageous for Babiš’s business.

For the Oath and Motorists coalition, the most prominent common themes of the elections were opposition to the Green Deal, particularly the planned ban on combustion-engine cars, as well as concerns about migration and certain other European policies. Despite this, the two parties differ. In the case of the Oath, its stance (not only towards the EU) is “softer.” Motorists cannot be considered representatives of hard Euroscepticism either – in their program, they clearly declare their affiliation with the EU (and NATO), but at the same time call for its internal transformation into a community of “self-confident and sovereign nation states that jointly strive only for what they agree on, but otherwise do not interfere in each other’s internal affairs.” Motorists are significantly more right-wing than Oath and declare themselves to be the only representatives of the true right, with many of their program points resembling those of the Civic Democratic Party in the 1990s.

As mentioned above, several populist parties are proponents of Czexit, including the SPD and Enough!, both of which demand a referendum on Czech membership in the EU. The SPD is a radical nativist, sovereigntist, anti-establishment party that promotes hard anti-immigrant, anti-Islam, and Eurosceptic stances. The party defines itself as “a patriotic and democratic movement” that struggles for “the independence and sovereignty of the Czech State”. According to the party, the EU poses a real threat to the existence of national states. The “European superstate” is considered undemocratic, imposing Islamisation through migration, and aiming at liquidating the nation state. The party rejects multiculturalism, which is perceived as a tool for Islamisation. In this regard, the SPD claims that Brussels corrupts national elites, who thus end up supporting immigration, a narrative typical of radical right-wing populist parties (cf. Mudde & Rovira Kaltwasser, 2018).

Enough! presents itself as a left-wing and patriotic movement. The KSČM, an essential part of the movement, has long been characterized by a populist narrative (even though the party is not usually characterized as populist), which applies both at the national and European level, about a narrow circle of elites who have usurped the entire country at the expense of the people. This vision has been adopted by the entire Enough! movement: “This state does not belong to a narrow elite; it belongs to you. And we will ensure that it is returned to you. The Enough! movement is the strong voice of the silenced majority!”. In its program for the 2024 European Parliament elections, it highlighted issues similar to those of other Eurosceptic populist parties – primarily the Green Deal, migration, rejection of the euro, and restrictions on freedom of expression by the EU.

As previously mentioned, the leading voice of Euroscepticism has traditionally been associated with the non-populist ODS. The party rejects the adoption of the euro, the strengthening of EU competences, and a large part of European policies. Although the party claims that “we belong to the West,” a backlash against several European trends, particularly in the cultural and environmental spheres, is evident (cf. Naxera, 2025b). Moreover, before the 2024 European elections, party leaders emphasized themes similar to those of populist opposition parties (backlash against the Green Deal, against adopting the euro, against migration, and against strengthening the EU’s powers). In many respects, the strongest party in the government before the 2025 national elections is closer to the populist opposition than to its coalition partners in its stance toward the EU. In fact, the party’s deputy chairman and leader of the 2024 EP candidates, Alexandr Vondra, has clearly stated that their program is almost entirely consistent with that of Motorists. After all, the party’s foreign partners include Eurosceptic parties such as Brothers of Italy and Law and Justice.

Every European crisis has the potential to foster Euroscepticism. In the case of Czechia, the migration crisis has had a significant impact in this regard, resulting in the most substantial decline in EU support among Czechs to date ( STEM, 2024) and reinforcing the position of Eurosceptic parties, a phenomenon also observable in other Central and Eastern European countries. Czechia was the exception when it came to Brexit. Hard Eurosceptics typically moderated their position towards the EU ( Hloušek & Havlík, 2024). The opposite has been proven in Czechia, where, for example, SPD perceived it as a signal for a Czech referendum ( Kaniok & Hloušek, 2018). The COVID-19 pandemic and the war in Ukraine have not significantly changed Eurosceptics’ position on Europe. However, they have given parties an opportunity to incorporate these issues into their discourse, thereby further consolidating their position ( Hloušek & Havlík, 2024; Kaniok & Hloušek, 2024). Donald Trump’s re-election was genuinely met with enthusiasm by Czech populist and Eurosceptic parties, as it meant another electoral victory for this style of politics. However, it quickly waned amid the debate over imposing tariffs.

## Responses to Euroscepticism and consequences

There are several mainstream pro-European parties in Czechia: two liberal parties – Mayors and Independents (STAN) and The Pirate Party, the conservative Christian Democrats (KDU-ČSL), and the liberal-conservative TOP 09 ( Sychra & Kratochvíl, 2021). These four center-right parties formed a coalition government with the ODS from 2021 to 2025 (the Pirates only until 2024). The coalition therefore had to balance the pro-European positions of these four parties (including support for the adoption of the euro, particularly evident in the case of STAN and the Pirates) with the Euroscepticism of the ODS, which was reflected in internal disagreements (for example, STAN distanced itself from the ODS on the issue of the European currency before the 2024 European Parliament elections). Pro-European parties maintained their position, while critiquing the Eurosceptic positions of populist parties. Interestingly enough, the critique was not as harsh in the case of ODS, as it was their coalition partner, and for some (TOP 09 and KDU-ČSL), their electoral partner. The other traditionally pro-European party, the Social Democrats (SOCDEM), has lost relevance (falling from 32% in 2006 to missing the 5% threshold in 2021). This has led them to join the hard Eurosceptic coalition, Enough!. This shift was, at least in part, an opportunistic move opposed by some pro-European members of the party. Some left the party as a result.

We can therefore assume that, with the exception of SOCDEM, the established mainstream parties are not giving up in response to the successes of Eurosceptic parties. The biggest shift towards Euroscepticism is thus the transformation of the populist ANO movement described above, which is also clearly driven by pragmatic reasons.

## Conclusion

Eurosceptic attitudes among political parties are relatively strong in the Czech Republic. Parties that could be described as hard or soft Eurosceptics currently have a clear majority, with some of them even openly calling for a so-called “Czexit.” This does not necessarily mean that Czexit is a real possibility, as only the SPD and Enough! are calling for it directly. One of them (Enough!) did not make it into the Chamber of Deputies at all, and the other (SPD), perhaps somewhat paradoxically, backed away from its pledge as a compromise made when forming a new government with other populist but soft-Eurosceptic parties, ANO and Motorists.

As previous research shows (cf. Naxera, 2025b), the distinction between Eurosceptic and pro-European parties is not necessarily related to that between populist and non-populist parties, since the ODS, a traditionally Eurosceptic party, is non-populist. Czechia thus serves as an example that the relationship between populism and Euroscepticism is not deterministic but rather a complex and dynamic process ( Andreadis & Vasilopoulou, 2026). Nevertheless, it can be concluded that Eurosceptic populism is on the rise in Czechia, particularly in its right-wing form, as evidenced by ANO’s growing Eurosceptic rhetoric and its current alliance with some far-right parties in the European Parliament.

## Ethics and consent

Ethical approval and consent were not required.

## Data Availability

No specific data besides sources cited in the manuscript were used.

## Footnotes

^1^
In February, 2026 Nikola Bartůšek (Oath and Motorists) parted ways with Patriots for Europe and is now a non-attached member. Antonín Staněk replaced Filip Turek, another MEP-elect for Oath and Motorists, after Turek was elected to the Chamber of Deputies in the national elections.
