# Supplementary material for: EUPopLink Country report - Czechia
Source: Open Res Eur. 2026 Jul 16;6:247. [Version 1] doi: 10.12688/openreseurope.23458.1 (PMC13560823; doi:10.12688/openreseurope.23458.1)
Supplement: Peer review history

# Reviewer report: Reviewer response for version 1

**DOI:** 10.21956/openreseurope.25387.r77746

**Published:** 2026-08-06

**Authors:** Nikola Petrović

**Affiliations:** 1 Institute for Social Research, Zagreb, Croatia

This is a well-written case study that deals with an important topic of the connection between populism and Euroscepticism in Czechia. However, it could benefit from addressing several issues in more depth.

 Firstly, it could delve deeper into the issue of populism and its connection with Euroscepticism. It would be interesting to explore whether early CEE Czech exceptionalism in Euroscepticism was influenced by strong populist tendencies in Czech society in the early 1990s. Mudde (2000) wrote about Havel’s antipolitics and the radical right populism of SPR-RSČ as an important early example of CEE populism.

 Secondly, the authors mention SPR-RSČ and Public Affairs as populist parties with which they do not engage with. However, dealing with them briefly could strengthen the paper in two ways. First, it could demonstrate the differences and similarities between the Eurosceptic populism of SPR-RSČ and contemporary forms of Eurosceptic populism. For instance, the prominent role of anti-communism in the case of Eurosceptic SPR-RSČ seems to be missing in new populist Eurosceptics. There is even a certain nostalgia towards socialist self-sufficiency that is used to develop anti-Western attitudes in SPD (Refer 1). Second, the non-Eurosceptic centrist populism of Public Affairs strengthens the paper’s conclusion that the relationship between populism and Euroscepticism is not as straightforward as it seems.

 Thirdly, the dynamism of Euroscepticism when it comes to power or in relation to governing parties has always been part of the Euroscepticism-populism nexus. It would be useful for the authors to engage with this dynamic. What did ODS’s softening of its Euroscepticism while in government mean for populist Eurosceptics? ODS is mentioned several times, but only as a consistent non-populist Eurosceptic voice in Czechia. It was argued that ODS became less Eurosceptic while in government, and was deemed as Eurorealist (Refer 2). The paper suggests that the war in Ukraine did not play an important role, but it could be argued that, as in the case of Brothers of Italy, it prompted the Eurosceptic party closer to the pro-EU mainstream.On the other hand, what does ANO’s coming to power in a post-Orbán environment mean for its populist Euroscepticism?

 Minor issues: Motorists emerging as a single-issue party should be mentioned.

 ‘conspiracy circles’ should be explained.

 In the case of ANO, ALDE should be mentioned, as it is a rare case of a liberal party transitioning to a radical right populist Eurosceptic party.

 References:

 Refer 1 (Beauduin et Al, 2024)

 Refer 2 (Cabada et Al, 2024)

 Refer 3 (Mudde et Al,2001)

Is the case presented with sufficient detail to be useful for teaching or other practitioners?

Yes

Is the work clearly and accurately presented and does it cite the current literature?

Yes

If applicable, is the statistical analysis and its interpretation appropriate?

Not applicable

Are all the source data underlying the results available to ensure full reproducibility?

No source data required

Are the conclusions drawn adequately supported by the results?

Yes

Is the background of the case’s history and progression described in sufficient detail?

Partly

Reviewer Expertise:

Euroscepticism, Europeanism, ideology

I confirm that I have read this submission and believe that I have an appropriate level of expertise to confirm that it is of an acceptable scientific standard.

## References

[1] Beauduin, A. (2024). The Czech Parliamentary Radical Right and the West: Harnessing Postsocialism to Anti-liberalism. Intersections. East European Journal of Society and Politics, 10(1).

[2] Cabada, L. (2024). The 2024 European elections in Czechia: A further strengthening of Euroscepticism. Politique européenne, 86(4), 70-77.

[3] Mudde, C. (2001). In the name of the peasantry, the proletariat, and the people: populisms in Eastern Europe. East European Politics and Societies, 15(01), 33-53.

# Reviewer report: Reviewer response for version 1

**DOI:** 10.21956/openreseurope.25387.r77743

**Published:** 2026-07-28

**Authors:** Claudiu Crăciun

**Affiliations:** 1 The National School of Political Science and Public Administration, București, Romania

The Report provides a brief but comprehensive overview of the positions of Czech parties on two ideological choices: populism and Euroscepticism. The Czech case has specific features: Czech parties are more Eurosceptic than those in other CEE countries and more inclined to adopt populist stances. However, this does not translate into hard Euroscepticism or into a popularly backed EU exit political strategy.

 Thus, a significant question is why Czech political and civil society was not mobilized around Eurosceptic stances, and perhaps instead around populist, anti-elite lines that help diffuse tension around the EU's role.

 Vrablikova's work can offer insights into the party-society continuum and how contention and grievances were structured in late transitional Czech politics. Another useful perspective would be to look at the interplay between Euroscepticism and populism from a territorial (and party) perspective. The Motorists provide an interesting example, with their transformation from a local to a national party.

 Second, the STAN party directly roots its identity in local politics, illustrating the possible avenues for articulating positions on the EU along the European-national-local continuum. A solid research agenda argues that populism and Euroscepticism cannot be understood without including the concept of space and place. Identities and grievances, including those related to the EU, are territorially defined and partially channeled.

 A recent overview of the connection is proposed by Dunin-Wasowicz and his colleagues (Dunin-Wasowicz et al., 2025) and is directly relevant to the Czech Republic as well. Populist and Eurosceptic voting (and mobilizations) have sub-national variations linked to socio-economic transformations. Parties shape their national outlook on the basis of local-national articulations of dissatisfaction and opposition, and the Czech party system could be a very relevant case in this respect. Overall, the strength of this report is that it presents populism and Euroscepticism not only as discursive choices but as potential strategies of relevant party actors whose imperative is to survive and gain real political influence.

Is the case presented with sufficient detail to be useful for teaching or other practitioners?

Yes

Is the work clearly and accurately presented and does it cite the current literature?

Yes

If applicable, is the statistical analysis and its interpretation appropriate?

Not applicable

Are all the source data underlying the results available to ensure full reproducibility?

Yes

Are the conclusions drawn adequately supported by the results?

Yes

Is the background of the case’s history and progression described in sufficient detail?

Yes

Reviewer Expertise:

Party politics, Euroscepticism, Populism, Social movements

I confirm that I have read this submission and believe that I have an appropriate level of expertise to confirm that it is of an acceptable scientific standard.

## References

[1] : Czech Republic—From Apathy to Mass Mobilisations, 1989–2022. 10.1007/978-3-031-77888-9_5109-135 10.1007/978-3-031-77888-9_5

[2] : Bridging the Divide Between Structural and Actor-Oriented Explanations of Populism: A Research Agenda for the Study of Populist Euroscepticism Through a Territorial Perspective. *Political Studies Review* .2025;23(2) : 10.1177/14789299241296796622-634 10.1177/14789299241296796
